# Many Things to Many People: The Diversity of Motivations for Joining Diasporic Organizations in the Global South

**DOI:** 10.1177/08997640241300515

**Published:** 2024-11-21

**Authors:** Miriam J. Anderson, Madeline F. Eskandari

**Affiliations:** 1 Toronto Metropolitan University, Ontario, Canada; 2 University of Toronto, Ontario, Canada

**Keywords:** diasporas, refugees, peace activism, transnational feminism, civil society organizations

## Abstract

Small, grassroots organizations in the Global South play an increasingly prominent role in political advocacy and service provision for displaced populations. Literature on both diasporic organizations and civil society organizations has largely focused on those based in the Global North, however. This article examines the formation of a transnational women’s organization—*Mouvement Inamahoro: Femmes et Filles pour la Paix et La Securité*—by refugees following Burundi’s 2015 electoral crisis. We focus on the diversity of motivations for individual members in founding and joining it based on 68 interviews conducted in 2019 and 2021. We find that members’ geographical location (Global North vs Global South) played a role in the motivations for membership. Our findings suggest a form of diasporic organization currently untheorized, but one relevant to understanding the influence of displaced groups in local, regional, and international politics.

## Introduction

Most refugees worldwide are hosted in a neighboring country to the one they fled (United Nations High Commissioner for Refugees [UNHCR], 2023). Accordingly, it is small, grassroots organizations in the Global South that play an increasingly prominent role in political advocacy and service provision for displaced populations (Milner & Klassen, 2021). However, literature on diasporas focuses overwhelmingly on those based in the Global North. The misalignment between the literature on diasporas and the reality within displaced communities limits our understanding of the pivotal roles played by diasporic organizations located in the Global South, which may have a membership that spans populations in both the Global North and South. This article examines a transnational diasporic women’s organization—*Mouvement Inamahoro: Femmes et Filles pour la Paix et la Securité*—founded in exile in Rwanda, predominantly by refugees, following Burundi’s 2015 electoral crisis. The organization has features of both a diaspora—since its members identify as Burundian and the vast majority live outside the country, and a transnational feminist network—due to its all-female membership and its explicit objective to advance women’s rights. *Mouvement Inamahoro* engages in humanitarian work and political advocacy, providing affective and material benefits to its members. It undertakes multiple activities, such as radio broadcasts aimed at the Burundian government and at Burundians; lobbying the Rwandan government and the UNHCR on behalf of Burundian refugees in Rwanda; training in leadership for women and girls; and mobilizing resources through members of the organization, based throughout Africa, Europe, and North America (Anderson & Eskandari, 2023, 2024).

This article focuses on the organization’s importance to the individuals who have founded and joined it based on 68 interviews (of 50 individual members) conducted in 2019 and 2021. In so doing, we uncover the complexity, diversity, and multifaceted nature of the motivations that drive *Inamahoro*’s members. Overall, we find that about 80% of members joined because they identified with the official objectives of *Inamahoro*. However, we find a bifurcation between members living in the Global North and those based in the Global South, regarding the relevance of sociability with those in the Global South much more likely to cite affective benefits related to sociability. Furthermore, the “myth of return”—which looms large in literature on diasporas (Koinova, 2012; Lyons, 2007; Orjuela, 2008)—was not a strong characteristic of diasporans living in the Global North. However, for diasporans in the Global South, the desire to return is more akin to an imperative than a “myth.” Diasporans “closer to home” in more precarious circumstances appear motivated by a very real need for change in their homeland, as opposed to idealizations disconnected from their own lived experiences.

This article is situated at the nexus of three bodies of literature: joiners and founders, diasporas, and transnational feminism. The literature on joiners and founders focusses on why individuals join and found voluntary associations. This body of work considers the salience of individuals’ attributes, incentives, and the import of social networks in determining the likelihood of one’s joining. The second literature examines diasporic organizations. It displays the multifaceted and multilayered objectives and activities of diasporas and has often focused on those based in the Global North. This lack of attention to organizations based in the Global South has meant a dearth of knowledge regarding the services these organizations provide and the composition of their membership. Furthermore, the literature has undertheorized the variation in motivations by geographical location and has left largely unexplored all-women’s diasporic organizations. The third body of literature focuses on transnational feminism and has examined the efforts of women’s cross-border activism. It has, to date, not examined the work of women of a single national identity. *Inamahoro* provides us the opportunity to examine the intersection of the three bodies of work shedding theoretical insight on all three.

*Inamahoro* formed in the wake of violence, with the mission to promote peace and security in Burundi. In response to Burundi’s President Pierre Nkurunziza’s seeking an unconstitutional third term, protests erupted to which government forces responded with violence (Daley & Popplewell, 2016, p. 649). Over 400,000 fled the country, seeking refuge throughout Africa, Europe, and North America, with a concentration of refugees in Rwanda and Uganda (Mbazumutima, 2023).

In 2021, *Inamahoro*’s members numbered about 60 Burundian women from a range of ages, ethnicities, political affiliations, and socioeconomic backgrounds, although a majority of its members are currently un/under employed, who live in exile throughout Africa, Europe, and North America with only about 10 members still residing in Burundi. Most of *Inamahoro*’s members remain “close to home” in East Africa—56% of the 50 we interviewed live in Rwanda, of whom 6 live in the Mahama Refugee Camp, and 19% live in Uganda. Only about 22% lived in Europe and North America.

*Inamahoro* brings together both Burundian women who had been politically active for decades and those who became engaged during and following the events of 2015.^1^ While a majority of these women did not hold positions in politics before becoming refugees, a small subset—4 of 50 interviewees—were former representatives of Burundi’s National Assembly and Senate, diplomats, officials of political parties, and judges. A number of the women were involved with prominent Burundian civil society organizations. Some of them had a role in the 1998 to 2000 Arusha peace negotiations (Anderson & Valade, 2023), and to our knowledge none of the interviewees held formal political positions in the Global North.

The article is structured as follows: we review the literatures on founders and joiners, on diasporic organizations, and on transnational feminist networks. We then outline our methods and examine and discuss the individual reasons for joining that *Inamahoro*’s members provided to us in semistructured interviews, contrasting the reasons for joining provided by members in the Global North with those provided by members remaining in East Africa. Finally, we offer some conclusions on the significance of these findings for the aforementioned literatures.

## Literature Review

### Founders and Joiners

The literature on joining is characterized by two approaches to explaining the formation and expansion of various organization types (Smith, 1994). The dominant approach examines the characteristics of individuals who form or join organizations, analyzing whether any particular characteristic or personal belief is associated with the likelihood to participate (Carman & Nesbit, 2013; Clary & Snyder, 1999; Compion, 2017; Lanero et al., 2017; Meier zu Selhausen, 2016; Smith, 1994). This literature has generally found that characteristics such as education, socioeconomic status, gender, and previous involvement in civil society influence individuals’ engagement.

In addition to characteristics, certain beliefs are associated with joining organizations—such as those consistent with the organization’s goals about how joining would benefit the individuals themselves or broader society (Clary & Snyder, 1999; García-Mainar & Marcuello, 2007; Monroe, 1998; Smith, 2000). Similarly, the literature on founders suggests that the founders’ beliefs are highly influential in the formation of nonprofit organizations. Specifically, a founder having strong beliefs about an issue and perceptions that the government or the market is failing “to address needs in the society” are dominant explanations for the founding of nonprofit organizations (Carman & Nesbit, 2013). With reference to both founders and joiners, David Horton Smith (2000) elaborates a typology of “incentives” motivating individuals to join grassroots associations: sociability, purposive, service, informational, developmental, utilitarian, charismatic, lobbying, and prestige.

In addition to individual characteristics and “incentives,” there are also numerous studies that analyze “organizational type” as determinative of joining in addition to individual characteristics (Gordon & Babchuk, 1959; Lansley, 1996; Williams & Ortega, 1986). For instance, Williams and Ortega (1986) find that characteristics typically associated with joining are only correlated with joining certain common voluntary organization types (p. 36). Thus, the organization’s structure and functions will also influence the composition of its membership.

Finally, a smaller branch of the literature on “founding” and “joining” extends beyond individual determinants of propensity for collective action and instead explores the role that social networks and context play. In addition to critiquing the focus on “individual-level variables,” these network-focused studies examine mobilization as a determinant of joining. Mobilization can refer to formal recruitment by an organization. However, much of the literature on mobilization has also looked at the role of preexisting online and offline social networks and how membership in these networks can result in being asked to join or cofound an organization (Campbell, 2013; Diani, 2015; Larson, 2021). For instance, Schnable documents the extent to which preexisting religious social networks in the United States were instrumental in the creation and expansion of grassroots international nongovernmental organizations (NGOs) operating in Africa (Schnable, 2021).

Campbell (2013) argues that the two dominant approaches to analyzing propensity for participation—individual-level explanations and social networks—are not mutually exclusive, as certain individual-level determinants of joining speak to the existence of a larger social network. Similarly, Schnable’s (2021) findings regarding religiosity influencing founding and joining speak both to individual characteristics and to group settings hospitable to collective action. Thus, given the ties between individual-level and network-level explanations for collective action, Campbell (2013) suggests that “the most compelling research on who participates will synthesize individual and social explanations” (p. 40).

### Diasporas

The term diaspora has broadened to include most displaced groups that maintain ties to a “homeland” rather than merely groups who left the homeland in the “distant past” (Délano & Gamlen, 2014). Diasporic organizations can influence politics in the “homeland” via advocacy, but can also often engage in service provision to members of the diaspora in the “host” country (Adamson, 2020; Bermudez, 2011; Betts & Jones, 2016; Bradley et al., 2019; Brinkerhoff, 2011; Brubaker, 2005; Faria, 2014; Henry & Mohan, 2003).

The literature on diasporas has extensively explored the motivations and actions of organizations and their individual members, which are multifaceted and multilayered (Koinova, 2018). Their activities span the gamut from leaders in exile destabilizing their home country (Krcmaric & Escribà-Folch, 2023), to financing terrorist groups (Piazza, 2018), to mobilizing for transitional justice (Orjuela, 2018), to building complex networks of exiles regionally and globally to affect politics and justice (Straussberger, 2022). Generally, scholarship on diasporas has found that diasporic organizations create “deterritorialized communities” (Shain & Barth, 2003), by engaging in processes of political identity formation independent of the “homeland” and by participating in service provision to assist members of the diaspora in the “host” country and those remaining in the “homeland” (Bermudez, 2011). Service provision is often via remittances by individual members or in some cases involves more complex fundraising efforts (Henry & Mohan, 2003).

In addition, many diasporic organizations attempt to politically influence the “host” country with ideas that formed within the diaspora (Adamson & Demetriou, 2007). This is often accomplished via lobbying in the “host” country, and collaborating with organizations or other actors remaining in the “homeland” (Al-Ali & Koser, 2002). A less frequent phenomenon observed in the literature is diasporas which support parties to armed conflict in the “homeland” such as actions taken by the Sri Lankan Tamil and Sinhalese diasporas (Fair, 2005). Conversely, diasporas often work to promote peace in the homeland (Orjuela, 2008). Although some observe that in the context of an authoritarian “homeland,” the diaspora is well placed to take political action by virtue of its independence (Kapur, 2014), others question the legitimacy of diasporic actors as representatives of those remaining in the “homeland” (Lyons, 2007).

Regardless of the desirability of diasporic political engagement, the literature generally has found that diasporic organizations engage politically in hopes of returning to a “peaceful” homeland or a homeland that favors one side of a conflict (Koinova, 2012; Lyons, 2007; Orjuela, 2008). This aspiration is one side of the multifaceted “myth of return” generally due to the group not actually having an intention to return by virtue of being far removed from the “homeland” and its perceived troubles. Because of this disconnect, efforts to improve the “homeland” may actually be undertaken to strengthen the diaspora as a community distinct from the actual country of origin (Al-Ali & Koser, 2002). Aspirations to “return” have also been characterized as a “myth” in cases where there is no recognized “homeland” of the displaced group akin to a state (Koinova, 2012). The concept has expanded to include not just actual return but “imagined” or “fantasy” returns (Bolognani, 2016; Oxfeld & Long, 2004) and to explore the multiple roles that visits home have (Miah, 2022). Within an increasingly globalized world, the concepts of home, journey, and return themselves have become blurred (King & Christou, 2011). Return is not necessarily an unchanging aspiration in the life of migrants, but is “reconsidered at times as a place of saviour and home, as well as a last resort or even an unwanted destination” (Bilgili, 2022, p. 38).

At the individual level of analysis, the literature has generally found two reasons motivating diasporas. First, elites are generally considered predisposed to act for change in the “homeland,” often to compensate for a sense of “status loss” in the “host” country (Betts & Jones, 2016; Guarnizo et al., 2003). Second, a sense of obligation often fuels diaspora activism for several reasons. Brinkerhoff (2011) observes that members of diasporas are motivated “by a sense of obligation or guilt, as they seek to reconcile their preference for the adopted country with their allegiance to a suffering place of origin” (p. 124). The sense of obligation can also stem from loved ones remaining in the “homeland” (Wong, 2006). In Africa specifically, the idea of “Ubuntu”—expected service to others—can prompt diaspora activism and aid (Compion, 2017; Fowler, 2022).

### Transnational Feminism

Literature on transnational feminist organizing illuminates why individuals, including members of diasporas, organize across borders via feminist organizations specifically. This scholarship has focused on feminist networks that operate in multiple states as opposed to vague ideas about “global sisterhood” (Conway, 2017; Desai, 2013; Grewal, 2005; Hughes et al., 2015; Mendoza, 2002). These studies explore the role “connectivities” across borders play in operationalizing feminist goals, such as a feminist peace and the participation of women in politics. Transnational feminist organizing can be defined as “women from three or more countries who mobilize for research, lobbying, advocacy and civil disobedience to protest gender injustice and promote women’s human rights, equality, and peace” (Valentine Moghadam as cited in Desai [2013, p. 429]). Ultimately, in the context of feminist diasporic organizations, the literature has found that the purposive goal of a feminist peace—both within the country of origin and in the host country—is an influential reason for joining. A feminist vision of peace is more than the mere absence of war and is steeped in ideas of substantive equality (Confortini, 2006; Davies et al., 2017; Sjoberg, 2010). It encompasses positive rights and freedoms such as access to food and education. Importantly, feminists conceptualize peace as being for everyone—including the most marginalized members of society (Olivius et al., 2022). This means that they reject the dichotomous idea that war and insecurity only exist during periods of active armed conflict and instead look to all forms of insecurity, including “nonspectacular” or “everyday” threats to peace such as hunger and inequality (Cockburn, 2004; Wibben, 2019; Wibben et al., 2019).

Nevertheless, the above-mentioned literatures do not quite capture the reasons why people found or join diasporic organizations in the Global South like *Inamahoro*. Although the political and economic contexts are highly influential determinants (Haddad, 2006), the literatures on diasporas and joining have maintained an overwhelming focus on organizations based in democracies in the Global North—mainly the United States (Brass et al., 2018). Exiled organizations based in the same region as the “homeland” such as *Inamahoro* generally cannot act in the same manner as organizations based in democracies out of the “homeland’s” reach (Adamson, 2020; Michaelsen, 2018). As Koinova (2012) observes, if members of a diaspora are faced with the real possibility of repression from the “homeland” or “unwanted return,” their political autonomy will be inherently limited. Per Koinova, differences in “positionality” can shape which members of diasporas can act and what they can do (p. 100). Similarly, Michaelsen (2018) documents the extent to which the internet has permitted the Iranian government to engage in “repression without borders” targeting dissidents abroad.

Given that our focus is on individual incentives for joining a diasporic organization, Smith’s (2000) typology provides a robust structure with which to analyze the responses of a diverse range of interviewees, with attention to their individual characteristics, beliefs, and social networks. Smith’s typology enables us to categorize these incentives into distinct yet interconnected categories while adapting it to the circumstances of displaced populations. This typology allows for a more structured comparison of motivations across different locations and social settings, enhancing our understanding of why individuals engage with diasporic organizations like *Inamahoro*.

## Method

Our findings are derived from a larger longitudinal research project, started in 2019, which documented *Inamahoro*’s activities to understand how a diasporic women’s peace organization operated when neither armed conflict nor formal peace talks were ongoing. The researchers, having hired a member of *Inamahoro*, utilizing research funds developed an archive of the activities and documents of the organization between 2015 and 2022, including press releases, screenshots of social media, advocacy letters, media programming transcripts, and internal governance documents.

The second major component of the research involved conducting interviews. We interviewed individuals at all levels of the organization, including the leadership, on various committees, and in diverse geographical locales. Members of the research team conducted 32 in-person interviews in Rwanda in 2019 and 36 via Zoom/WhatsApp in 2021 (since some respondents were interviewed twice, we have a total of 50 unique interviewees in our sample). Relevant to this article, we asked interviewees when they joined, why they joined, their previous involvement in civil society/formal politics, and questions on their sociodemographic status.

We first transcribed the interviews and then coded interviewee responses according to Smith’s (2000, p. 96) nine-category typology of joining incentives, summarized in Table 1.

**Table 1.** Summary of Smith’s Incentive Typology.

Incentive type	Description
Sociability	Opportunity to socialize with group’s members
Purposive	Desire to contribute to achieving the association’s goals
Service	Satisfaction from providing informal/formal service to members and nonmembers
Informational	Opportunity to gain new information, knowledge, and skills
Developmental	Achieving personal growth from a volunteer role
Utilitarian	Obtaining economic returns, including money, discounts
Charismatic	Opportunity to associate with charismatic leaders of the association
Lobbying	Participating in the association’s attempts to influence policy and politics
Prestige	Desire to earn prestige through membership in the organization

Although *Inamahoro* is not a “grassroots association” according to Smith’s definition since grassroots associations have a small geographical scope, subsequent contributions applying this typology of “incentives” to diasporic organizations indicate its relevance as a framework through which members’ reasons for joining in the Global North and South can be easily compared. For instance, in the context of a diasporic organization, Brainard and Brinkerhoff (2004, p. 35) conceptualize the “sociability” incentive as thicker than a mere desire to socialize, including the desire for community belonging. Given the importance of both the characteristics and beliefs of joiners in the literature, we focus on geographic location while also coding reasons for joining using Smith’s typology, which differentiates between categories of “incentives” regarding the organization, its goals, and its activities.

The research team operationalized the framework and coded interviewee responses as follows. We coded a member as indicating “sociability” if they mentioned the affiliative benefits of belonging to the organization, such as: seeking solidarity among the Burundian members or wishing to develop friendships with them. Our working definition may be broader than Smith’s since the level of need for community and belonging among a newly displaced population is more acute than that of individuals living in a more stable environment. We coded for a “purposive” incentive if the member’s reason for joining aligned with the Movement’s official objectives, which include working for peace and security in Burundi and promoting the political participation of women and girls.^2^ We coded for “service” when members stated that they had wished to “contribute” to the work of the Movement in some way. Members’ incentives for joining were coded as “informational” in cases where members said they wished to learn about or better understand what was going on in Burundi. We coded a member’s incentives as “developmental” if they said that they wanted to learn new skills through membership in *Inamahoro*. A “utilitarian” coding was applied if a member had said they wanted to benefit materially from membership. We coded a member’s incentives as “charismatic” if they said that they wanted specifically to work with and/or associate with particular members of the Movement. A member was coded as having a “lobbying” incentive if they stated that they wanted to influence politics and policy through their participation in *Inamahoro*. Finally, we coded members as motivated by “prestige” incentives if they had indicated that they sought membership in *Inamahoro* to benefit reputationally.

## Results

In this section, we highlight the variation among *Inamahoro’s* members regarding their incentives to join. We find that the most prominent reason for joining *Inamahoro* was purposive across both the members in the Global South and the Global North, by which we mean that their motivations align with one or more of *Inamahoro*’s objectives. We note that two respondents did not provide any codable incentives. Table 2 presents the percentages calculated by number of respondents.

**Table 2.** Incentives Reported and % (by Number of Interviewees).

Incentives	Total	North	South
Purposive	39 (81.3%)	8 (80.0%)	31 (81.6%)
Sociability	20 (41.7%)	2 (20.0%)	18 (47.4%)
Service	13 (27.1%)	2 (20.0%)	11 (28.9%)
Informational	5 (10.4%)	2 (20.0%)	3 (7.9%)
Charismatic	3 (6.3%)	1 (10.0%)	2 (5.3%)
Developmental	2 (4.2%)	0 (0.0%)	2 (5.3%)
Utilitarian	2 (4.2%)	0 (0.0%)	2 (5.3%)
Respondents’ total	48	10	38

We also find a significant minority of members, predominantly in the Global South, reported joining for reasons which could be classified as “sociability,” meaning that they sought the affiliative benefits of belonging to the organization. Although, as will be discussed below, this is a thicker conception of sociability than theorized by Smith. Fewer members sought membership for reasons related to obtaining information, personal development, the desire to serve others, or to obtain material benefits that membership might provide.

When coding for purposive incentives, we also found that these related to two major areas: a general desire to see democracy return to Burundi and a second focused on feminist ideals. Thirteen women cited both when stating their purposive incentives, 8 mentioned the feminist ones only, and 18 just the democracy one (see Table 3).

**Table 3.** Breakdown of Reported Purposive Incentives.

Purposive by type	Total	North	South
Democracy	18 (46.2%)	3 (9.7%)	15 (48.4%)
Democracy and feminist	13 (33.3%)	3 (9.7%)	10 (32.3%)
Feminist	8 (20.5%)	2 (6.5%)	6 (32.3%)
	39	8	31

Before exploring the difference that geographic location makes, we first examine major reasons for joining that were common among most members, regardless of their geographical location: a desire to respond to major political events in Burundi, previous involvement in civil society, already knowing founding members of the organization who asked them to join, and interest in *Inamahoro*’s objectives and activities.

## The Multifaceted Nature of Joining

### Shared Reasons for Joining

#### Initial Formation of Mouvement Inamahoro: Politicization in Response to Protests and Exile

Since *Inamahoro* formed following Burundi’s electoral crisis in 2015, it is unsurprising that most members—regardless of whether they were in Burundi at the time of the crisis—characterized their decision to found/join the organization at least partly as a reaction to the political turmoil in Burundi and the mass exodus that it catalyzed. In fact, the desire to join a peace organization stems from perceptions that the Burundian government was not safeguarding peace and was creating insecurity. Many members phrased their desire to join *Inamahoro* to “bring peace and security back to Burundi.” We coded this motivation as purposive, as members sought to participate in the official objectives of *Inamahoro*.

Nevertheless, in addition to a general desire to promote peace during a time of insecurity, there were also specific aspects of the 2015 crisis that galvanized members to join. For instance, many members were inspired after seeing—either firsthand or at a distance—the involvement of women in the 2015 protests. These protests erupted after former President Pierre Nkurunziza made clear he would seek an unconstitutional third term. Some of *Inamahoro*’s membership met one another while demonstrating in Burundi—a salient example of the links between individual and network explanations for joining. For instance, one member in exile in Rwanda explained that she met the founding members of *Inamahoro* in 2015 at the protests in Burundi unintentionally. She had been working in an office nearby and inspired by the women’s efforts; she decided to buy them mangos to eat.^3^ In fact, several members including *Inamahoro*’s President Dr. Marie Louise Baricako also described the formation of the organization as the product of women who were involved in or inspired by the 2015 protests feeling “compelled to act and raise their voice.”^4,5^ One member living in Belgium explained that she felt called to join the “brave Burundian women who had decided to take their destiny into their owns hands, to participate in all areas of national life and to raise their voice to denounce crimes and human rights violations.”^6^

*Inamahoro* is an ethnically diverse organization explicitly advocating against interethnic violence. Many members remarked that, after witnessing a diverse set of Burundian women demonstrating together, they were inspired to join a more formalized organization. For instance, one member living in Canada partially attributed her desire to join *Inamahoro* to having heard about

the mass demonstration that took place in Bujumbura that brought together women of all social classes, of all political classes, of all ethnic groups, to protest against human rights violations.^7^

Perhaps, seeing mass demonstrations of unity on the streets of a divided country made members feel willing and capable to engage in more formalized collective action.

Finally, the insecurity and violence associated with fleeing Burundi, regardless of whether experienced firsthand or through accounts by others, ranked as the most significant incentive for joining. Exile clearly animates this entire contribution—given that in subsequent sections we will illustrate how isolation due to exile and hopes of returning to Burundi were key reasons why members in the Global South joined *Inamahoro*. For instance, one member residing in the Mahama refugee camp in Rwanda claimed that witnessing women raped and tortured as they fled Burundi in 2015 motivated her to join an organization whose stated mission was to advocate for a broad peace in Burundi and help women:

There were a lot of people, a lot of ladies, who were raped when we were fleeing. Many ladies who were traumatized. Raped girls with a whole host of problems: health problems, mental health problems. So, I saw the Movement’s aspirations and then I applied for membership . . . Given that we were fleeing from security issues, I ought to join everyone who will talk about peace, who will talk about security, because here at the camp, there were a lot of problems. The people who fled also had many problems.^8^

Even members who were not in Burundi in 2015 were inspired to join the organization largely because of learning of insecurities associated with exile. One member in Europe noted:

Because of the war in 2015, many women have been in really hard conditions, most of the time they fled the country empty-handed with children. And if we join the Movement, we are able to know exactly what is happening since the Movement is close to the women and refugee families. Since the Movement is in direct contact with them, we know how to help them through the Movement.^9^

Those living as refugees in East Africa had a more acute incentive to address the needs of a vulnerable population receiving minimal state support. As one member living in the Mahama refugee camp observed,

Bad circumstances united the members of the Movement. As you know, bad circumstances unite women very much. More than happy circumstances. Because, see how our Movement was born. It was born in exile, by exiled people. There are people who get lost and who need other people to lift them up. So, *Mouvement Inamahoro* is the Movement that has come together to lift up the weakest, to come to the aid of those who have problems, and to be the bandage for the wounds of other ladies who were raped, who knew major problems in our country, who are widows, orphans, all of that. Yes. That is what unites us the most. Being the bandage of the most wounded.^10^

### Previous Involvement in Civil Society and Social Networks

Members from both groups also cited their previous involvement in civil society and politics as a reason for joining *Inamahoro.* We coded these responses as being incentivized by a desire to be of “service.” Based on the responses, both explanations ring true—many members felt that their experience gave them both the skillset to be of help in a diasporic peace organization and the desire to partake in it. Some members phrased this in terms of civil society giving them certain values, such as being “committed to protecting the rule of law.”^11^ Others considered participating in *Inamahoro* as their way of contributing their skills to bring peace and security back to Burundi. One member living in Rwanda who had been involved in both politics and civil society in Burundi prior to 2015 explained the feeling of being willing and capable of joining by virtue of previous experience as follows:

I was always, I would say, an active woman. So, I have always been in women’s associations. And so, if we say, “we’re going to do something for us.” I would say “very well,” because . . . what I offer is my experience, my knowledge, my time, my determination, my engagement. So I believe that I have everything to offer. I have time, I am a person with experience at the level of civil society, maybe at the political level, at the level of everyone who can be useful concerning Burundi. I would like to give a lot and I am willing to give, especially commitment because I believe I am committed to the cause of women.^12^

Like her, a number of *Inamahoro*’s members were involved in Burundian politics and civil society prior to 2015 and expressed similar sentiments about how these experiences prompted them to join. However, *Inamahoro* has many members with no experience in politics or civil society prior to 2015 who also cited their desire to contribute their skills to peacebuilding as a reason for joining. For instance, many members have law degrees from Burundi and felt that this experience could benefit the organization.

In addition, the majority of members remarked that they were asked to join the organization by founding members whom they had known prior to 2015. Several others characterized *Inamahoro* as a remobilization of previous networks of Burundian feminist activists that began during the 2015 crisis and then formalized in exile. For instance, *Inamahoro*’s President Dr. Baricako described the circumstances of how *Inamahoro* was founded as follows:

The Movement started as a spontaneous action from women, some of them who were already in women’s organizations, already engaged in women’s advocacy. Others were young women who believed and thought they should involve themselves because their country was in crisis so other some women felt like joining the others so they started in the demonstrations of May 2015 but the biggest part of them was building on what they had been doing even before.

### Members “Closer to Home”: Isolation, Seeking a “New Family,” and the Myth of Return

The majority of *Inamahoro*’s members are refugees who left Burundi following the 2015 crisis but remained in East Africa, mainly in Rwanda and Uganda. The experience of life as refugees in the Global South influenced members’ accounts of how and why they joined. In fact, most members located in East Africa joined at least partially due to rather personal experiences of isolation, which we coded as being an incentive based on a thick conception of “sociability.” This understanding of “sociability” involves both the desire for socialization initially discussed by Smith and the desire for support in a community setting as theorized in the literature. One member located in Rwanda explained that she joined the organization “to end my solitude and fight for women’s rights with other women.”^13^ Another explained that she was so lonely that she joined to be able to engage with other women: “It’s very personal and very selfish. But to be able to speak. That was it. To be able to find myself with people.”^14^ Members recounted personal stories of depression following the traumatic experience of fleeing Burundi. They explained that leaving behind their lives in Burundi gave them a strong desire to find “a new family” and that *Inamahoro* was able to fill this role:

From the first day that I joined the Movement, first of all I gained a family. Why a family? Because I had come to Rwanda, I had fled my country, I had not come with my family. I stayed at home every day. I had no friends; I had no family members. So, when I joined the Movement, I started feeling fulfilled. I met other people, other Burundian women, with whom we speak the same language, with whom we discuss peace and security, with whom we discuss women’s problems, with whom we discuss the problems in Burundi, the crisis in Burundi, what we can do . . . So, that is something very important when you are far from your loved ones, when you are abroad, when you are a refugee.^15^

Members spoke positively about joining a “new family” composed entirely of women with whom they could share a “common struggle.” According to Dr. Baricako, the organization has explicitly aimed to fill the role of a “new family” because of its members’ experiences of exile. Many members reported that they stand in solidarity together when one of them is struggling. For instance, speaking of *Inamahoro*’s capacity-building activities, one member observed that “if a woman feels isolated and has low self-esteem, in the Movement we teach them to fight for peace and security.”^16^ Evidently, the isolation associated with exile facilitated the formation and expansion of *Inamahoro*. Finally, members “closer to home” cited aspirations to return to Burundi as a reason for joining *Inamahoro*, which we coded as a “purposive” incentive.

None of *Inamahoro*’s members in the Global North expressed any desire to return to Burundi whereas most of *Inamahoro*’s members in Rwanda and Uganda claimed that at least part of the reason why they joined was due to their aspirations to return to Burundi. For instance, one member in Uganda remarked: “To return, I need to be in the Movement and fight with others for the return of peace in my country.”

### Members in the Global North: Obligation to Join

As previously discussed, a significant minority of *Inamahoro*’s membership reside in the Global North—Europe and North America. These include members who have not lived in Burundi (or Africa) for most of their lives and also members who lived in Burundi until the 2015 crisis. While Burundi’s 2015 crisis and mass exile were a major motivation in joining *Inamahoro*, we found substantial difference in other motivations to join between members residing in the two regions.

What was especially notable about the reasons for joining that were unique to this segment of the membership was their striking similarity to how the literature on diasporic organizations has conceptualized joining. This is somewhat unsurprising given that this literature is often concerned with organizations based in the Global North (Nguyen, 2020; Wong, 2006). As previously discussed, we found that members in the Global North joined due to a general desire to influence homeland politics following the 2015 crisis.

However, what was surprising was the extent to which this group’s reasons for joining went beyond a general desire to promote political change in Burundi and actually resembled more specific claims. For instance, many of this group of members expressed that at least part of why they joined *Inamahoro* was due to a sense of guilt or obligation—either due to having loved ones remaining in Burundi or due to their preference for the country that they currently resided in. Several of *Inamahoro*’s members’ accounts of their joining substantiate this claim. For instance, when asked why she joined the organization, one member who had recently come to Canada as a refugee remarked that “I truly would like to have a country where each of the compatriots are free and enjoy the rights of citizens like in other countries such as Canada.”^17^

Furthermore, in many cases, the desire to become a member of the organization was rooted in familial obligation. Unsurprisingly then, family ties were a reason for joining expressed by members in the Global North. For instance, one member of the organization who had resided in Belgium since childhood explained:

Well, I live very far from my country already. Since very long ago. But I still have frequent contact with my family who is in Burundi. And because I always worry a lot for my family, for my country. And so, I really keep myself very informed, very regularly of everything that goes on there. And I told myself that it was a way to raise awareness a bit more around me on what is happening and to see also how I can intervene or help a bit.^18^

Unlike members residing in the Global South, no members of *Inamahoro* residing in the Global North expressed any desire to return to Burundi. This was true regardless of how long they had resided outside of the Global South. Perhaps, this is the case because all of *Inamahoro*’s members in the Global North are in constant communication with members in the Global South via WhatsApp and other means. In fact, some told us that they received hundreds of messages daily about current events in Burundi and in the Burundian refugee community. In addition to being informed, members in the Global North also play a large role in *Inamahoro*’s advocacy. Members told us that they regularly attend advocacy meetings, record radio messages, participate in lobbying activities, and more. This is largely because being public faces of the organization is much safer in the Global North.

## Discussion

Four major elements emerge from our research, which speak to the extant literature. First, *Inamahoro* supports its members in countering the lack of institutional support felt by refugees. However, the organization also provides important personal and social connections to its members, including offering a vehicle for the sense of obligation felt by members toward their families still in Burundi. Finally, we have an interesting take on the “myth of return.”

In addition to contributing to the literatures on founders and joiners and diasporas, understanding the formation and growth of organizations in the Global South is significant, given the increasing role of nonstate actors in the areas of advocacy and service provision as a result of the shrinking of the neoliberal state (Brass, 2022). As a transnational, diasporic women’s peace organization operating primarily in the Global South, *Inamahoro* affects the lives of many via its efforts to operationalize a feminist peace. The unprecedented importance that even small grassroots organizations have in the daily lives of ordinary people means that who participates and how are more relevant than ever. Moreover, membership in organizations is often associated with future participation in activism and formal politics (Brass, 2022; Walker, 2008). For instance, feminist groups similar to *Inamahoro* have been characterized as unifying forces who are willing and capable to work with their so-called enemies to facilitate peace and social change (Anderson, 1999, 2000). Thus, why members participate and the functions of the organization that they participate in are relevant to understanding how and why change happens. The formation of *Inamahoro* during a time of crisis corresponds with the literature’s emphasis on government failure as a reason for collective action.

Among the members of an organization like *Inamahoro*, most of whom are refugees in very vulnerable conditions, sociability needs are much more acute than in Global North grassroots organizations. In terms of the personal and sociability dimensions, we find that women joined *Inamahoro* partially because of certain skills and values resulting from previous involvement in collective action. This is ultimately unsurprising and aligns with a central claim in the literature on joiners, that individuals gain skills and values via education and civil society involvement, predisposing them to join. We also believe that prior involvement in this case works both as an individual-level and as a network-level explanation for joining. Various responses note that joining represented something akin to “finding a family,” which fits well with Smith’s (1994, 2000) argument that the affiliative benefits of membership can create powerful, longer relationships and friendships among members and that, in some cases, some or all of a member’s close friendships may be drawn from the organization.

The third element relates to the literature on the sense of obligation that members of diasporas—especially women—feel to assist their families “back home,” usually in the form of remittances, but also to Brinkerhoff’s (2011, p. 124) point that members of diasporas act due to a “sense of obligation” prompted by a desire to “reconcile their preference for the adopted country with their allegiance to a suffering PO [place of origin].” This sense of obligation emerges very clearly from our responses from *Inamahoro*’s members in the Global North.

The sense of obligation found in the literature on diasporas is akin to the spirit of solidarity driving transnational feminist networks. *Inamahoro* resembles a transnational feminist network in that its goals are explicitly feminist and its membership spans multiple countries. In *Inamahoro*’s case, however, all of its members are Burundian. It is still remarkably diverse, however, in that its members are drawn from multiple ethnicities, socioeconomic backgrounds, political affiliations, and countries of residence. It is able to mobilize resources through cross-border linkages, drawing on the relative influence of members closer to centers of power. During COVID lockdowns in Rwanda, for example, *Inamahoro* successfully mobilized its transnational membership to raise over $17,000 to provide humanitarian assistance to Burundian refugees (Anderson and Eskandari, 2023).

The final reflection is about how the myth of return is interpreted and lived by *Inamahoro* members. First, we found that some prominent claims about why diasporas in the Global North take political action were not cited as reasons for joining by any of *Inamahoro*’s Global North members. For instance, part of the literature on diasporas suggests that the “myth of return” is a prominent reason for joining—referring to the idea that diasporas act to influence the homeland in hopes of returning to it. The literature has attributed this desire to return to the status loss that is often experienced by members of diasporas in the host country (Koinova, 2012; Orjuela, 2008). Some have also attributed the prevalence of the “myth of return” to the fact that members of diasporas residing in the Global North are “out of touch” and consequently do not understand daily life in the country of origin (Koinova, 2012).

Conversely, members living in East Africa were adamant that a key goal was to return to a (democratic) Burundi. This is arguably a facet of the “myth of return” distinct from those theorized in the literature documenting diasporas, given that members in Rwanda and Uganda are aware of ongoing problems in Burundi and have many contacts there and yet still express a desire to return. In fact, many face the prospect of a potential “unwanted return” per Koinova (2012), making improving the homeland an imperative as opposed to a “myth.” Rather than being fuelled by a lack of true knowledge of Burundian politics, their desire to return is rooted in the idea that *Inamahoro* as an organization is capable of aiding in the restoration of peace to Burundi.

Perhaps, this is because of its advocacy and initiatives to build the capacity of women and girls to participate in formal politics. As well, it could be because several of its members held prominent positions in Burundian politics and civil society prior to 2015, including in the Arusha peace process. Irrespective of the reason for their faith in *Inamahoro*, examining the reasons for joining of members located in East Africa indicates that the realities of exile—isolation and missing the homeland—are highly influential in the formation of diasporic organizations that are based “closer to home.” When members of a diaspora are within the state’s reach and return is a real possibility, the “myth” of return becomes less of a myth and more of a concrete goal.

As King and Christou (2011) noted, a more globalized world, where affordable and instant video, text, and voice communication is accessible, may have had an effect on the myth of return, especially considering the ability of members in the Global North to have constant and effective information from Burundi. This “virtual proximity” to Burundi may help to explain why we find this discrepancy between the literature and the goals of *Inamahoro*’s *Global North members.*

## Conclusion

This study contributes to the literature on diasporas’ account of why diasporic organizations form during a prolonged exile. Our findings contribute to the idea that the “myth of return” is a complex concept and not necessarily a driving motivation of organization members in the Global North. Whether or not they are well informed about the situation in the homeland, their motivations for joining were overwhelmingly purposive. We have suggested that this is due to *Inamahoro*’s unique membership attributes—namely, that it is mostly composed of members “closer to home” who communicate frequently with those in the Global North about the realities of life in Burundi. We also find that members in the Global South joined due to a desire to return to a peaceful Burundi. However, rather than being a “myth,” the prospect of return is akin to an “imperative” for *Inamahoro*’s members in the Global South, who view joining a peace organization such as *Inamahoro* to be an essential step toward improving the situation in Burundi prior to a potential unwanted return. Evidently, our findings highlight the nuances of the “myth of return,” demonstrating how it can be extended to situations where members of diasporas seek to influence homeland politics due to perceptions of return as inevitable, regardless of whether it is desired.

We also find that *Inamahoro*—a diasporic organization—has many features in common with a transnational feminist network, where explicitly feminist objectives are sought among a regionally diverse membership. Feminist objectives, the spirit of solidarity, and the mobilizing of resources are key to the functioning of *Inamahoro*, where bridges are built across different ethnicities, socioeconomic backgrounds, political affiliations, and countries of residence. This broadens what might be considered a transnational feminist network to include diasporic organizations committed to feminist objectives.

We also find that exile—both the reaction to the political events associated with it and the personal isolation that it created—is a powerful catalyst for joining. For members in the Global South, a thicker conception of Smith’s concept of “sociability”—including a genuine need for socialization, a sense of community, and a social safety net—figured prominently in explanations for joining.

In sum, we find that whether members resided in the Global North or Global South influenced their reason for joining *Inamahoro.* However, our findings suggest that reaction to political events, social networks, previous involvement in civil society, and agreement with the organization’s objectives as reasons for founding and joining were highly influential regardless of the location of the individual member.

More broadly, we find that those members geographically closer to Burundi had a more diverse set of incentives for joining *Inamahoro*. This is likely because the material conditions faced by refugees in the Global South are far more precarious than those who obtain refugee status or citizenship in the Global North.

Finally, this study provides evidence of more diversity within a diasporic organization than previously envisaged. Most research on diasporas has focused on organizations whose membership and activities are based in the Global North. By examining the various reasons for founding/joining *Inamahoro*—an organization led in the Global South, with members living in exile close to their country of origin—we see that diasporic organizations are a place for women to engage in politics, build community, and support humanitarian efforts. Women who joined were diverse in terms of their previous involvement in politics/civil society, when/if they left their country of origin, and their place of exile. We suspect that there are organizations like *Inamahoro*, but which go unnoticed because of the challenges in researching them and the biases of where politics are usually conducted.

Avenues for future research should include a more in-depth look at how the technologies used by *Inamahoro* members, and that are available to other diasporic organizations with far-flung memberships, affect their activities, in particular in the Global South. Throughout the time we followed *Inamahoro*, a number of structural changes occurred because of members moving from the Global South to the Global North, new members joining, and a rift in the organization. This calls for further research to track the dynamic nature of like organizations in precarious and evolving circumstances. Organizations like *Inamahoro* serve to fill deep and varied needs among refugee populations. Understanding what the organization means to individual members demonstrates how small, yet impactful, organizations are many things to many people.

## Notes

1. For an analysis of Burundi’s 2015 crisis, see Bouka (2017) and Vandeginste (2015).

2. “(1) Contribute to the restoration and consolidation of peace and security through the mechanisms of prevention, conflict management, and protection of citizens; (2) Promote the effective and equitable political contribution of women and girls. (3) [sic]; (4) Participate in the process of peaceful conflict resolution and of peace consolidation in an effective and equitable manner according to Resolution 1325 of the United Nations Security Council and to all other subsequent resolutions on women, peace, security, and peace consolidation; (5) Contribute to the restoration of a rule of law that is respectful of the Constitution and of Arusha Agreement for Peace and Reconciliation; (6) Contribute to the strengthening of the capacities of women and girls within the political, juridical, social, economic, environmental and technological domains for their development and empowerment; (7) Promote the safeguarding of the sociocultural values that constitute the pillars of Burundian society; (8) Constitute a think tank to influence the formulation and implementation of policies, strategies and programmes” Mouvement des femmes et filles pour la paix et la sécurité au Burundi (2017). Statuts du mouvement des femmes et filles pour la paix et la sécurité au Burundi “MFFPS” en sigle. In Mouvement des femmes et filles pour la paix et la sécurité au Burundi (Ed.).

3. Interview 19, 2019.

4. Interview 26, 2019.

5. Interview 11, 2019.

6. Interview 27, 2021.

7. Interview 4, 2019.

8. Interview 7, 2021.

9. Interviewee 13 2021.

10. Interview 7, 2021.

11. Interview 14, 2019.

12. Interview 10, 2021.

13. Interview 9, 2021.

14. Interview 10, 2021.

15. Interview 6, 2021.

16. Interview 20, 2021.

17. Interview 5, 2019.

18. Interview 1, 2021.
